# Telemedication for Opioid Use Disorder: A New Approach for Treatment

**DOI:** 10.1089/tmr.2023.0036

**Published:** 2023-11-08

**Authors:** Rebecca A. Georgiadis, Paige Sable, Daniel Rosen

**Affiliations:** ^1^ Univerisity of Pittsburgh Medical Center Health System, School of Medicine, University of Pittsburgh, Pittsburgh, Pennsylvania, USA.; ^2^ Department of Social Work, University of Pittsburgh School of Social Work, Pittsburgh, Pennsylvania, USA.

**Keywords:** MOUD, telehealth for MOUD, COVID-19 effects, opioid treatment

## Abstract

**Objective:**

This study aimed to determine the effect that the transition to incorporating remote care had on patients in medication for opioid use disorder (MOUD) treatment and to identify benefits and gaps in services due to this transition.

**Materials and Methods:**

Treatment data on patients receiving buprenorphine through the MOUD program were collected using electronic medical records. A 28-month window was created and encompassed three time periods: in-person MOUD prepandemic, fully remote/telehealth for MOUD (tele-MOUD), and hybrid services (combination of tele-MOUD and in-person care).

**Results:**

Rates of reported illicit drug use were consistent across all three time periods, with no statistically significant differences. Attendance at MOUD appointments and urine drug screen completion trended downward during fully remote care but approached prepandemic levels during hybrid services.

**Discussion:**

Tele-MOUD presents opportunities for improving treatment of patients with opioid use disorder, and hybrid models should continue to be adopted, evaluated, and reimbursed by insurances.

## Introduction

The COVID-19 pandemic exacerbated the devastating effects of the opioid epidemic on an already vulnerable population.^1,2^ To mitigate the spread of COVID-19, many health care services transitioned to remote services to limit risk of infection and fill a need in care of chronic conditions during the pandemic, including opioid use disorder (OUD).^3^ In response to the need for infection control among individuals with OUDs, Federal lawmakers invoked an exception to the 2008 Ryan Haight Act, permitting the use of telehealth for buprenorphine induction and allowing Medicaid reimbursement for telehealth appointments for addiction treatments.^4^

As a result, medications for opioid use disorder (MOUD) programs, which was previously exclusively an in-person program with face-to-face evaluations, abruptly transitioned to a telehealth platform to operate remotely at the onset of the pandemic. This marked the first-time remote platforms for the delivery of treatment services, also known as telehealth for MOUD (tele-MOUD), were utilized.^5^ Although these services were implemented to meet the needs of patients during the pandemic, tele-MOUD also addressed many barriers to care that had previously hindered individuals from accessing treatment for OUD, including transportation challenges, employment obligations, or education responsibilities.^6^

This study aimed to examine the impact of transitioning to remote care (tele-MOUD) on patients receiving MOUD treatment at an urban federally qualified health center (FQHC). Furthermore, we investigated the potential benefits of accessing tele-MOUD for individuals with OUDs, as well as identified gaps in services that emerged with the transition to tele-MOUD. Finally, we offer recommendations for the development of viable and effective tele-MOUD programs.

## Methods

From Fall 2019 to Spring 2022, we conducted three electronic medical record reviews at different time points to analyze the buprenorphine treatment provided to patients as part of the MOUD program at an urban FQHC in the midwest. These reviews covered three distinct periods of clinic policies on in-person treatment within a 28-month window, representing in-person MOUD (prepandemic: October 2019–February 2020), fully remote care (fully tele-MOUD: March 2020–October 2020), and hybrid services (mix of tele-MOUD and in-person care: November 2020–February 2022).

These time periods were chosen due to the abrupt closure of the clinic to all in-person visits in March 2020 mandated by state and county health officials. At this time, all MOUD patients were rapidly transitioned to exclusively telehealth services. In October 2020, the clinic reopened to in-person care with MOUD patients remaining fully remote until November 2020, at which point patients were permitted to attend both in-person and virtual care appointments. We employed descriptive and bivariate statistical analyses to identify any differences between these time periods. The University of Pittsburgh Institutional Review Board approved this study.

### Measures

The FQHC implemented regular urine drug screens (UDSs) as a requirement for patients enrolled in the MOUD program. Before the COVID-19 pandemic, UDSs were conducted on site at the FQHC. However, when services shifted to remote care, patients were instructed to attend virtual appointments using a secure video platform and to visit the nearest community-based diagnostic clinic for UDS completion. To assess the impact of this change, travel times from patients' home addresses to local diagnostic clinics were measured and compared with travel times to the FQHC using public transportation routes on Google Maps.^7^

The analysis of UDS results served as a measure of abstinence from illicit substances. The screened substances included unprescribed opioids (such as heroin, fentanyl, and oxycodone), unprescribed benzodiazepines, unprescribed stimulants (including methamphetamines, Adderall, and cocaine), and unprescribed gabapentin. In addition, deidentified data were collected on demographics, and treatment protocol adherence and retention metrics (including UDS completion, self-reported drug use, and appointment attendance) as indicators of engagement with the MOUD program.

## Results

### Demographics

A total of 62 patients were identified as part of the MOUD program during the study period, with 37% receiving treatment for the entire duration. The prepandemic program (October 2019–February 2020) had 33 patients, the fully remote care program (March 2020–October 2020) had 34 patients, and the hybrid services program (November 2020–February 2022) had 41 patients.

The age of the patients ranged from 24 to 65 years, with a median age of 41 years. In terms of gender, 42% were male, 56% were female, and 2% identified as nonbinary. As for race, 77% were white/Caucasian, 2% were African American, 2% were American Indian, and 19% did not specify their race.

### Adherence and retention

Regarding adherence and retention, the average travel time by bus from a patients' homes to the FQHC was 52 min, with a minimum of 12 min and a maximum of 104 min. The average travel time from patients' homes to the nearest local diagnostic clinic through bus was 24 min, ranging from 4 to 44 min, representing an average reduction of 53.8% in travel time.

Table 1 presents the rates of UDS across different time points, comparing positive UDS results and the reported use of illicit substances. The trends of reported illicit substance use and positive UDSs were similar throughout all time periods, with no statistically significant differences observed. However, there was a statistically significant change in UDS completion rates, with 100% completion during in-person sessions, 38.4% completion during fully remote care, and 83.7% completion during hybrid services.

**Table 1.** Urine Drug Screen Results and Reported Drug Use

	In-person MOUD (*n* = 33), %	Fully remote (*n* = 34), %	Hybrid services (*n* = 41), %
Reported illicit drug use	26.7	18.2	22.4
Positive UDS for illicit substances	27.6	20.9	23.6
Percent of patients who had UDSs	100 (FQHC)	38.4^*^ (diagnostic clinic)	83.7^**^ (Hybrid)

^*^ Statistically significant than in-person, *p* < 0.001.

^**^ Statistically significant than fully remote, *p* < 0.005.

FQHC, federally qualified health center; MOUD, medication for opioid use disorder; UDS, urine drug screen.

Attendance was also used as a measure of adherence and retention. The average number of missed appointments during in-person MOUD was 1.70, which increased to 1.90 with fully remote services and decreased to 1.69 with hybrid services. However, there were no statistically significant differences in the number of missed appointments across the different time periods. Notably, the majority of missed appointments in all three time periods were for therapy appointments.

## Discussion

The hybrid model, which allowed patients to receive care remotely through a HIPAA-compliant video program combined with in-person visits, demonstrated improved adherence in both UDS completion and attendance compared with a fully remote model. It achieved levels like those observed in fully in-person care. However, despite local diagnostic clinics being, on average, closer to the patients' residences, the percentage of patients undergoing drug screening at these clinics was significantly lower (*p* < 0.01) than in both fully in-person care and the hybrid model that offered both in-person and remote care. Furthermore, only 5% of patients who completed UDSs during hybrid care chose to visit a local diagnostic clinic for this purpose.

Several factors may contribute to this discrepancy in UDS adherence and the preference for conducting UDSs at the FQHC. First, during the government-mandated shutdowns in the early stages of the COVID-19 pandemic, there was a significant amount of uncertainty and fear associated with venturing out in public. This could have discouraged patients from visiting external locations, such as local diagnostic clinics. In addition, although diagnostic clinics may be physically closer to patients' residences, many of these clinics have limited operating hours specifically designated for UDS completion, which can be viewed as a stigmatizing practice when compared with the availability of other services.

The transition to remote care effectively addressed pre-existing barriers to MOUD treatment and maintained steady enrollment in care. However, this shift also introduced new obstacles to MOUD treatment. Many individuals encountered challenges related to the availability of reliable hardware devices to access the telehealth platform and limited access to stable internet connections. Furthermore, completing the UDSs was complicated by the restricted operating hours of local diagnostic clinics.

Both practitioners and patients faced technical difficulties when initially adapting to telehealth platforms, which affected the seamless delivery of services. In addition, patients often experienced a lack of privacy within their homes during telehealth appointments. Nevertheless, hybrid models that leveraged telehealth approaches provided patients with greater flexibility to adapt treatments to their schedules and demonstrated higher effectiveness in promoting treatment engagement than fully remote MOUD approaches.

A limitation of this study was that during the initial phase of the pandemic and subsequent shutdowns, patients resorted to various methods to remotely interact with clinic staff. As a result, before the establishment of secure internet connections and dependable technology, certain patients had to rely on telephonic means to communicate with the staff. In addition, marijuana was excluded as an illicit substance from the analysis.

Medical marijuana was legalized in Pennsylvania in 2018 and became available to qualifying patients, with some patients in the study obtaining medical marijuana cards, indicating their authorized use of marijuana. Finally, the sample size of this study is small, including only 62 patients from one FQHC. This along with the upheaval of the pandemic and transitory nature of the patient population limited the ability to conduct a more detailed analysis of the handful of patients who received care at the FQHC during all three time periods.

## Conclusion/Implications for Policy

Through exceptions to existing regulations and reimbursement policies, implemented in response to the COVID-19 pandemic, telehealth has provided health care providers with opportunities to engage a larger number of individuals in care. However, the sudden shift to this new care model has required policy makers and practitioners to swiftly develop programs and guidelines for a vulnerable patient population. Tele-MOUD presents various advantages, such as offering new perspectives into patients' lives, reducing barriers to accessing care, and enabling patients to have a say in the mode of their treatment. It is recommended that tele-MOUD programs be intentionally designed to shape patient expectations for hybrid programs, thereby enhancing patient experiences and treatment adherence.

A hybrid model of tele-MOUD offers a means for patients to customize their care based on their specific needs. For patients seeking flexibility and convenience, tele-MOUD provides the opportunity to incorporate more frequent tele-MOUD appointments into their care plan. To facilitate this, patient contracts can be employed, utilizing future tele-MOUD visits as an incentive for attending in-person appointments and allowing practitioners to require in-person visits when necessary. In addition to continuing Medicaid reimbursement for tele-MOUD, it is crucial to conduct regular analyses of programming and metrics to support ongoing quality improvement.

Future research should incorporate exploration of demographic, social determinants of health, disease profile, and treatment history in individual-level analyses of tele-MOUD use. This will help ensure that program goals are being met and that patient outcomes leveraging the full benefits of tele-MOUD can be achieved and tailored to individual patient needs.

## Authorship Contribution Statement

R.A.G. contributed to conceptualization, data curation, formal analysis, methodology, writing—original draft, and writing—review and editing. D.R. was in charge of conceptualization, methodology, supervision, and writing—review and editing. P.S. took care of writing—review and editing.

## Abbreviations Used

FQHC: federally qualified health center
MOUD: medication for opioid use disorder
OUD: opioid use disorder
Tele-MOUD: telehealth for MOUD
UDSs: urine drug screens
